# Vestibular Rehabilitation Improves Gait Quality and Activities of Daily Living in People with Severe Traumatic Brain Injury: A Randomized Clinical Trial

**DOI:** 10.3390/s22218553

**Published:** 2022-11-06

**Authors:** Marco Tramontano, Valeria Belluscio, Elena Bergamini, Giulia Allevi, Sara De Angelis, Giorgia Verdecchia, Rita Formisano, Giuseppe Vannozzi, Maria Gabriella Buzzi

**Affiliations:** 1 IRCCS Santa Lucia Foundation, Via Ardeatina 306, 00179 Roma, Italy; 2 Department of Movement, Human and Health Sciences, University of Rome “Foro Italico”, Piazza Lauro de Bosis 15, 00135 Roma, Italy

**Keywords:** inertial sensors, wearables, movement analysis, biomechanics, neurorehabilitation, sTBI, balance, locomotion, smoothness, RCT

## Abstract

Neurorehabilitation research in patients with traumatic brain injury (TBI) showed how vestibular rehabilitation (VR) treatments positively affect concussion-related symptoms, but no studies have been carried out in patients with severe TBI (sTBI) during post-acute intensive neurorehabilitation. We aimed at testing this effect by combining sensor-based gait analysis and clinical scales assessment. We hypothesized that integrating VR in post-acute neurorehabilitation training might improve gait quality and activity of daily living (ADL) in sTBI patients. A two-arm, single-blind randomized controlled trial with 8 weeks of follow-up was performed including thirty sTBI inpatients that underwent an 8-week rehabilitation program including either a VR or a conventional program. Gait quality parameters were obtained using body-mounted magneto-inertial sensors during instrumented linear and curvilinear walking tests. A 4X2 mixed model ANOVA was used to investigate session–group interactions and main effects. Patients undergoing VR exhibited improvements in ADL, showing early improvements in clinical scores. Sensor-based assessment of curvilinear pathways highlighted significant VR-related improvements in gait smoothness over time (*p* < 0.05), whereas both treatments exhibited distinct improvements in gait quality. Integrating VR in conventional neurorehabilitation is a suitable strategy to improve gait smoothness and ADL in sTBI patients. Instrumented protocols are further promoted as an additional measure to quantify the efficacy of neurorehabilitation treatments.

## 1. Introduction

Traumatic brain injury (TBI) is considered a major cause of mortality and long-term physical, cognitive, and behavioural disability in young adults, especially in high-income countries [1,2,3,4]. When the Glasgow Coma Scale (GCS) score (in the acute phase) is 8 or less [5], TBI is categorized as severe (sTBI). Lower quality of life is caused by disability following sTBI, which restricts daily activities and social interactions [6,7,8,9,10,11]. Balance and gait issues are frequently evident in sTBI patients [12,13], which increase their risk of falling and have a severe impact on their ability to perform activities of daily living (ADLs), their quality of life, and their ability to reintegrate into society [14,15]. From a clinical point of view, locomotor evaluation allows a quantification of gait quality; wearable devices, such as the inertial measurement units (IMUs), permit the quantitative estimation of walking parameters such as walking speed, stride frequency, and stride length either in ecological environments (i.e., hospital, clinic) or in real-life scenarios [16]. 

However, the cited spatio-temporal scores provide partial information about any existing limitation regarding dynamic postural stability and gait smoothness [17,18]. Furthermore, the assessment of gait and balance usually is only focused on straight walking tests, such as the 10 m, 2 min, or 6 min walk test, or the Timed Up and Go test, which do not adequately reflect mobility function during daily life. Recently, more ecological tasks have been developed and used in patients with neurological disorders to assess biomechanical parameters that are related to walking stability, symmetry, and smoothness during turning in clockwise and counterclockwise directions [19,20,21] and stepping blindfolded [22]. These instrumental evaluations could also discriminate between different levels of severity within the same cohort of patients [20,21].

Therefore, a quantitative and postural-related assessment using gait quality indices during straight and curved walking conditions could be appropriate for patients with sTBI [20]. Indeed, an instrumented-based assessment, integrated with clinical scales and questionnaires, allows quantitative and objective discrimination among different levels of walking abilities and an exploration of the relationship between the estimated gait quality indices and the clinical scale scores. This integrated approach has already been used to assess motor changes following vestibular rehabilitation (VR) training in stroke patients [23], which is a safe dynamic patient-centred physical therapy. VR includes a combination of different therapeutic modalities aimed at promoting gaze and postural stability in patients with neurological disorders [23]. VR can improve static and dynamic balance, fatigue, gait, and activities of daily living in different ages and neurological conditions [23,24,25,26,27]. Only a small number of studies have examined the effects of VR on concussion-related symptoms and vertigo in patients with TBI to date [28,29], while no clinical trials have been carried out on people with sTBI during the neurorehabilitation hospitalization period.

We hypothesized that standard neurorehabilitation training should include VR strategies to improve balance and postural stability in patients who have no vestibular syndrome modulating the vestibular brain network and sensory–motor integration.

Based upon the hypothesis that neurorehabilitation training during post-acute rehabilitation including VR might improve gait, dynamic stability, and consequently the ADL in patients with sTBI, the present study aimed at first at investigating VR effects on gait and balance evaluated by a combination of instrumental and clinical scales assessment, and secondly at evaluating the effects of VR on ADLs. The results of the present study could highlight the added value of VR treatments by observing the relevant clinical and kinematic changes induced. This evidence would represent an added value in the management of the patient with sTBI, supporting the choice of the best integrated and personalized rehabilitative strategy.

## 2. Materials and Methods

### 2.1. Study Design

**Figure 1:**
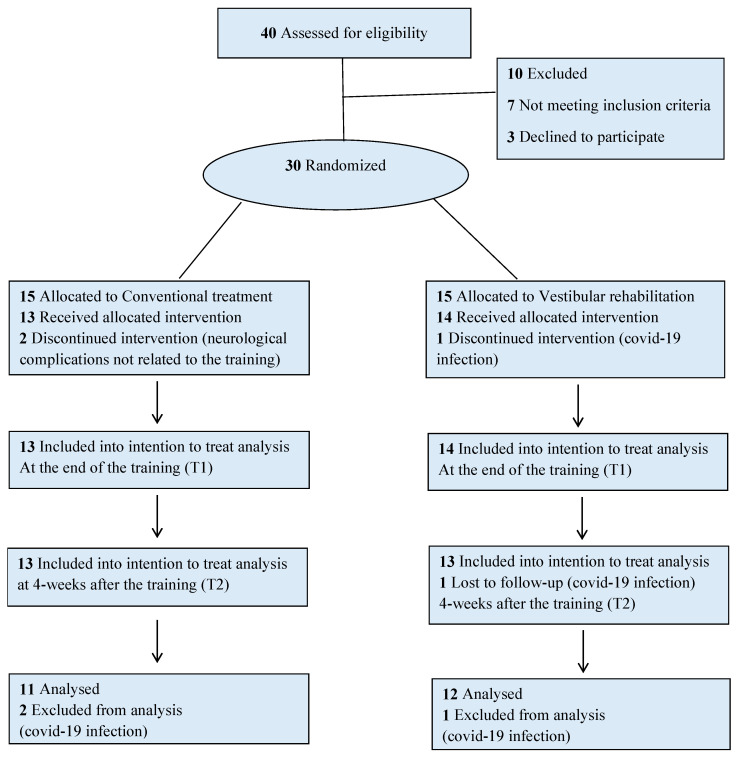
Flow diagram for randomization of patients with severe traumatic brain injury enrolled in the study.

This single-blind randomized controlled trial (Figure 1) had 8 weeks of follow-up as previously recommended in similar RCTs [25]. The Consolidated Standards of Reporting Trials (CONSORT) were followed. This trial was approved by the Local Ethics Committee of Fondazione Santa Lucia (Rome, IT) with the protocol number CE/PROG.700, and all participants were included in the study after providing their informed consent. The trial was registered before enrolment on ClinicalTrials.gov with the ID number NCT04415580. Based on the inclusion and exclusion criteria, a researcher who was not participating in the intervention sessions determined whether the patients were eligible to participate. Participants were randomized into one of two groups: a VR group (VRg) and a conventional rehabilitation group (CRg). The two subgroups were homogeneous with respect to demographic characteristics, anthropometry, and clinical status.

### 2.2. Participants 

Thirty sTBI inpatients were enrolled based on consecutive sampling within a six-month time window. This sample size met the minimum requirement set by an a priori power analysis for nonparametric between-group comparisons conducted on preliminary data (α = 0.05; β = 0.8; ES = 0.6) [30]. According to this sample size estimation procedure, each group should have had at least 13 patients. All participants were at their first neurorehabilitation admission since trauma. Almost all the patients selected suffered from sTBI as a consequence of a traffic accident, whereas two people suffered from sTBI due to a fall off a horse. Inclusion criteria were: aged between 15 and 65 years, Level of Cognitive Functioning (LCF) ≥ 7 [31]. Prior traumatic brain injury, cognitive deficits affecting comprehension of task instructions (Mini-Mental State Examination score of 24), severe unilateral spatial neglect (diagnosed using a battery of tests including the Letter Cancellation Test, Barrage Test, Sentence Reading Test, and Wundt–Jastrow Area Illusion Test), and severe aphasia, the presence of other neurological and psychiatric diseases, and the presence of orthopaedic or cardiac comorbidities that would limit participation in the experimental and conventional training were the exclusion criteria. 

Characteristics of participants at baseline who received intervention are reported in Table 1.

### 2.3. Interventions 

During their hospitalization, the enrolled patients underwent 12 individual sessions of neurorehabilitation, 3 days per week/4 weeks. Each session lasted 20 min. Two physiotherapists with at least 3 years of experience in VR with neurological disorders performed the training. The allocated interventions were performed as an add-on to the conventional neurorehabilitation [32] based on exercises in accordance with the individual rehabilitation protocols.

### 2.4. Vestibular Rehabilitation (VRg Only)

VR consisted of two different exercises for gaze stability (GS) and for dynamic postural stability (DPS). For the GS exercises, patients held their gaze on a firm target (VORx1), under physiotherapist supervision, during active horizontal and vertical head movements (one minute for each axis). GS exercises were carried out for no more than 10 min including a quick rest period and were performed under different conditions, i.e., seated, standing, and during a step on the spot. 

DPS exercises consisted of a step on the spot on a foam cushion 5 cm in height. Once the patient was in a stable posture and blindfolded, he/she performed a 1 min step on the spot and 1 more minute for each clockwise rotation at 90° 180°, and 270°. The same procedure was carried out at for a total of four minutes. If the patient inadvertently rotated left/right or moved forward/backward while stepping, the physiotherapist asked them to move back to their original position using verbal cues (e.g., “you are turning left/right” and “you are moving forward/backward”) [26,33,34]. The maximum exercise duration was 5 min, including quick rest periods. Immediately after preparation (1 min walk on a treadmill with eyes opened using a preferred walking speed), patients were blindfolded and asked to walk on the treadmill without the support of their hands for 4 min. In the case they changed direction, the physiotherapist asked them to maintain an appropriate position using verbal cues as above. The maximum exercise duration was 5 min, including warm-up and quick rest periods. 

### 2.5. Conventional Balance Rehabilitation (CRg only)

Conventional balance rehabilitation was focused on trunk stabilization and consisted of three exercises. Patients were asked to maintain their balance seated blindfolded on a Bobath ball for 5 min. A physiotherapist supported them in keeping the right position. Afterwards, they had to maintain balance in a standing position on a Freeman board for 5 min and transfer their body weight in a standing position using parallel bars for 10 min [35].

### 2.6. Blinding Randomization

The randomization was performed by an independent person who was not in charge of deciding whether patients were eligible. A computer-generated randomization list was used to produce block randomization using a block size. The researcher in charge of randomization stored the list in a safe online repository. Following the initial evaluation, the participant was handed a sealed envelope with the name of their assigned intervention group, which had been produced by a research assistant.

### 2.7. Clinical Outcome Measures

At enrolment, clinical and demographic data were collected. An examiner blinded to allocation assessed primary and secondary outcomes from baseline (T0) at 4 weeks of training (T1), at 4 weeks after the end of the training (T2), and after 8 weeks after the end of the training (T3). The primary outcome measure was the Dynamic Gait Index Scoring Form (DGI) to assess a subject’s ability to modify their gait in response to changing task demands. DGI consists of eight items rated from 0 to 3 (0 = severely impaired; 3 = normal performance), yielding a maximum score of 24 points. A score lower than 19 points is associated with a gait impairment risk of fall [36,37]. Secondary outcome measures were: the Berg Balance Scale (BBS), which measures 14 different tasks related to balance and postural control. The BBS is scored from 0 to 4, with 0 indicating that the subject is unable to perform the task and 4 indicating that the subject fully meets the most difficult criteria required for the task [38]; the Community Balance and Mobility Scale (CB&M) was used to assess specific aspects of balance and mobility that are necessary for independent functioning within the community [39]; the Dizziness Handicap Inventory (DHI) was used to evaluate self-perceived activity limitation and restriction resulting from dizziness; a sensor-based instrumental assessment was used to evaluate spatiotemporal gait parameters and gait quality indices (dynamic stability, symmetry, and smoothness) [40]. The Activities-Specific Balance Confidence Scale (ABC) and Community Integration Questionnaire (CIQ) were used to evaluate changes in the activities of daily living from baseline to 8 weeks after the end of training.

### 2.8. Instrumental Assessment

Two examiners blinded to patients’ allocation performed the instrumental assessment at baseline (T0) at 4 weeks of training (T1), at 4 weeks after the end of the training (T2), and 8 weeks after the end of the training (T3). All participants were asked to perform two different locomotion tasks: a linear 10 m walk test (10 mWT) and a curvilinear figure 8 walk test (F8WT). During the 10 mWT, participants were asked to walk on a straight 14 m-long walkway three times. Hence, in order to assess steady-state walking, only the middle 10 m (marked with tape on the floor) were considered for further analyses. For the F8WT, the participants were asked to walk a figure-8-shaped path marked with tape on the floor [20], consisting of two circles 1.66 m (5.44 ft) in diameter each. The task was performed three times in the counterclockwise direction. Both linear and curved tasks were randomly performed at participants’ preferred walking pace. 

### 2.9. Gait quality Instrumental Assessment

Each participant was given five synchronized IMUs (128Hz, Opal, APDM, Portland, OR, USA) to measure upper-body stability while completing the two locomotor tasks (10 mWT and F8WT). The IMUs were placed on the occipital cranium bone near the lambdoid suture of the head (H), the centre of the sternum (S), and the L4/L5 level, just above the pelvis (P). For step and stride segmentation, the other two IMUs were placed on both shanks, just above the lateral malleoli. Each IMU was made up of tri-axial accelerometers, gyroscopes, and magnetometers. It was attached to the participant’s body with Velcro straps, and each participant wore a swim cap with a specially designed compartment for the head IMU.

Several quantitative parameters in the spatiotemporal, stability, and smoothness domains were obtained for the two walking tasks, as follows:

Spatiotemporal: (*i*) Average walking speed (WS) as the ratio between total distance and time to complete the test; (*ii*) average stride duration (SD) as the ratio between time to complete the test and the number of strides; (*iii*) average stride frequency (SF) as the total number of strides divided by the time needed to complete the test. The number of strides was automatically obtained through a peak detection algorithm on the ML angular velocity signals measured by the two IMUs on the shanks [41]; 

Stability: Normalized Root Mean Square (*nRMS*) of the acceleration measured at pelvis, trunk, and head levels. The values were obtained for the AP, ML, and CC signal components as well as the magnitude value. High nRMS values have been associated with higher levels of acceleration, and hence, decreased stability [42]; 

Smoothness: LDLJa and LDLJv parameters were calculated from the linear acceleration and angular velocity components, respectively, as measured at the pelvis level [43]; lower LDLJ values have been associated with a higher level of unsmoothness of a translational/rotational movement. LDLJa and LDLJv rely on variations in accelerations and angular velocities, respectively, during the execution of the movement to quantify smoothness. With reference to LDLJv, the parameter is defined as follows:

$$\mathrm{LDLJv}≜-\ln\left(\frac{{\left(t_{2}-t_{1}\right)}^{3}}{v_{\mathrm{peak}}^{2}}\int_{t_{1}}^{t_{2}}{\left|\left|\frac{d^{2}}{{\mathrm{dt}}^{2}}v\left(t\right)\right|\right|}_{2}{}^{2}\mathrm{dt}\;\right)\;;$$

$$v_{\mathrm{peak}}≜\underset{t\in \left[t_{1},t_{2}\right]}{\max}{\left|\left|v\left(t\right)\right|\right|}_{2}$$

where **v**(t) represents the angular velocity of the movement in the time domain; t_1_ and t_2_ represent the beginning and end of the movement, respectively [43].

### 2.10. Statistical Analysis

Statistical analysis was carried out using SPSS (v.28, IBM Corp, Armonk, NY, USA). Normality of data distribution was assessed through the Shapiro–Wilk test (*p* > 0.05). In the case of skewed distribution, spatiotemporal and IMU-based data were transformed using Tukey’s ladder of powers.

A mixed-design analysis of variance model (4 × 2 ANOVA) was used to examine differences between sessions (T0, T1, T2, and T3) and group conditions (VRg vs. CRg). Tukey’s HSD test was used for post hoc comparisons. As most of the clinical scales’ data were not normally distributed, a nonparametric analysis was performed; specifically, the Friedman test and the Wilcoxon signed-ranks test were used for the within-subjects comparison for both groups at times T1–T0, T2–T0, and T3–T0. The level of significance was set to α = 0.05, adjusted using Bonferroni correction for multiple comparisons. 

## 3. Results

### 3.1. Clinical Assessment

Of the 30 initially recruited participants, 3 of them discontinued during the treatment period for reasons not related to the study; therefore, 27 patients completed treatments (Figure 1). Statistical analysis was performed using the data of twenty-seven patients who completed the evaluation at T1 (VRg = 14; CRg = 13), twenty-six patients who completed the evaluation at T2 (VRg = 13; CRg = 13), and twenty-three patients who completed the evaluation at T3 (VRg = 12; CRg = 11). Table 2 shows the clinical scores of the VRg and CRg at different times (T0; T1; T2; T3). 

Both groups showed a significant improvement in BBS, DGI, CB&M, and DHI scores in the within-subjects comparison over time. Specifically, the results of the Wilcoxon signed-ranks test highlight a significant improvement in BBS and DHI in both groups at T1, T2, and T3. The VRg showed a significant improvement in all three assessment times in the CB&M as well, while the CRg showed an improvement only 8 weeks after the end of the training (T3). Furthermore, only the VRg showed a significant improvement in the ABC and CIQ scores over the assessment sessions. Additionally, a significant between-group difference was found in the ABC and CIQ score.

For the sake of clarity, the within-group analysis results are related to comparisons T2 vs. T1, T2 vs. T3, and T3 vs. T1. Only patients treated with VR had improved ADLs as revealed by a pronounced increase in the mean (±SD) Activities-Specific Balance Confidence Scale score (baseline: 74.1 (±11.8); 8 weeks after treatment: 84.2 (±15.1); *p* = 0.04) and Community Integration Questionnaire score (baseline: 12.3 (±9.9); 8 weeks after treatment: 12.9 (±4.4); *p* = 0.01). 

### 3.2. Instrumental Assessment

The IMU-based assessment of linear walking during the 10MWT showed a significant main effect of the session on SF (F_1.483,16.314_ = 8.581, *p* = 0.005) and SD (F_1.424,15.660_ = 9.012, *p* = 0.005), pointing out a difference between T0 and T1, T0 and T3 and T2 and T3 (*p* < 0.05). Trunk and head RMS presented significant main effects of session on the AP direction (F_3,33_ > 4.232, *p* < 0.05) with progressively decreasing RMS values from T0 to T3, which were significantly different between the initial and the final assessment session. A main effect of the session was also highlighted for LDLJv-AP (F_3,33_ = 5.965, *p* = 0.002), showing a difference in gait smoothness between the initial and the final assessment (*p* < 0.05).

**Figure 2:**
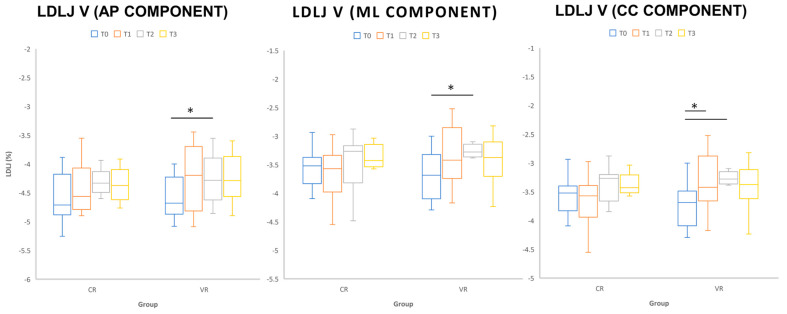
Whiskers’ plots of LDLJv smoothness parameter in the AP, ML, and CC components as obtained during figure 8 walk test in the different T0, T1, T2, and T3 sessions. Horizontal lines and asterisks indicate significant statistical differences (*p* < 0.05).

Group-driven interactions emerged when considering the instrumental assessment of curvilinear walking during the counterclockwise F8WT. The smoothness parameter LDLJv in the three directions pointed out a distinctive trend for the VR group (F_3,33_ > 7.274, *p* < 0.05), with significant differences between T0 and T1 and T0 and T2 (*p* < 0.05, see Figure 2).

The main effects of the session were widely observed in all the investigated subdomains of interest: spatio-temporal, stability, and smoothness. Table 3 summarizes all the observed main effects of the session.

## 4. Discussion

Our study shows that VR, when integrated in a neurorehabilitation program, improves all clinical scale scores of gait and balance performance as well as a conventional balance program. Interestingly, only patients who performed VR improved in terms of smoothness of gait and ADLs, as demonstrated by the analysis of curvilinear walking tasks and by the increase of the ABC and CIQ scores. 

From a clinical point of view, it is important to note that only the VRg showed a statistically significant improvement of the activities of daily living assessed 8 weeks after the end of the training, underlining the possible long-lasting effects of VR training compared to the conventional rehabilitation in patients with sTBI. Furthermore, the VRg showed a significant improvement in all the three assessment times in the CB&M as well, while the CRg showed an improvement only 8 weeks at the end of the training (T3). While this observation will surely require additional research to be confirmed, it may be hypothesized that VR training could promote earlier motor ability changes with respect to conventional rehabilitation, potentially facilitating a quick recovery.

The improvement in the instrumental qualitative parameters during dynamic and more challenging tasks is a clear result of our RCT study. Indeed, during the F8WT, significant differences were found between T0–T1 and between T0–T2 in all LDLJv smoothness components, suggesting that patients who performed more dynamic training were able to perform smoother curved paths. LDLJv is a smoothness parameter that represents an important additional metric to quantify the quality of rotational movement, representing postural responses due to treatments. The evaluation of smoothness is a useful index in neurorehabilitation because it could quantify motor recovery [44,45] and predict level of motor independence [46,47]. The improvement in LDLJv values during our curvilinear walking test suggests that VR training could be a valuable rehabilitative strategy to improve dynamic motor abilities while walking along a curved path at the end of the training and at follow-up. According to the existing literature [48,49], curved paths require more abilities in postural adaptations (with respect to straight walking) in order to fit the biomechanical constraints imposed by the linear and rotational dynamics of turning. Indeed, people with neurological disorders might encounter difficulties in managing the interplay between balance control and centre of mass progression when turning [20]. Balance and gait are complex multi-factorial systems. The weighting of the sensory inputs that continually control balance and gait is likely influenced by environmental factors and varies depending on the motor activity that the individual is engaged in [50,51]. For these reasons, the improvement found during the curvilinear task underlined the importance of implementing conventional neurorehabilitation with a task-specific approach, such as vestibular strategies, to facilitate the integration of all components (visual, vestibular, and proprioceptive) to ensure postural control in both static and dynamic conditions.

From an applied biomechanics perspective, this work promotes the adoption of integrated instrumented approaches to motor performance assessment. The abundance of main effects for session, in both instrumented locomotion tasks, highlights how the IMU-based biomarkers could support the evaluation of the efficacy of the rehabilitative treatment, whether it is conventional or vestibular. The added value of our instrumented protocol is in the fact that we can go far beyond a global collective score (as happens in the clinical tests), giving the clinician the opportunity to objectively examine subtle improvements in gait quality from different domain perspectives (such as gait stability and smoothness, for instance).

From a neurorehabilitative point of view, our results could help with the management of patients with sTBI for two important reasons: (i) gait and balance instrumental evaluation should be performed during more ecological and challenging contexts that consider curved walking as a condition more representative of real-life scenarios; (ii) a neurorehabilitation program could be tailored according to the results of instrumental assessments [52]. In this respect, our work is in line with very recent trends observed in the literature that progressively propose the joint use of wearables to objectify rehabilitation-driven movement changes and the adoption of motor tasks involving rotations to challenge the musculoskeletal system in motion. This approach is preferrable when aiming at identifying postural instability and could be also prospectively implemented into software applications that calculate IMU-based parameters in real-time and with a low computational cost [53].

Our results are innovative because we combined sensor-based gait quality evaluation, dynamic training (VR), and clinical scale scores in a novel multimodal method. As a result, patients who saw a notable increase in ADL activity also experienced a greater improvement in gait smoothness. It is reasonable to hypothesize that VR programs focused on increasing dynamic postural adjustments might reduce unsmooth movements with changes that are not easily detected by traditional evaluation techniques. Nonetheless, sensor-based assessment is confirmed to be a useful support in detecting any subtle improvements in gait quality elicited by either the conventional or our innovative vestibular training. 

## 5. Limitations

We are aware that the current study presents some limitations. First of all, we had many dropouts along the course of the study due to COVID-19 infections, potentially increasing Type-II error in the last assessment session (T3). As for our sample, the mean age is similar to that used in previous studies carried out on patients with severe TBI [20,54], but further studies could also evaluate the potential effect of sex and age as confounding factors on the clinical outcomes in this population.

Concerning the follow-up period, we did not use a specific algorithm to choose a period of 8 weeks for the follow-up; we rather considered this time window as appropriate to better understand the post-trial effects and the maintenance of gains outside the hospital. Indeed, this time period of follow-up is consistent with a previous clinical trial aimed at investigating the effects of VR in highly disabled patients on their activities of daily living after discharge [25].

Another limitation is the absence of an instrumental evaluation of the vestibular reflexes’ functions [55,56], but, even in a probable absence of specific vestibular damage for the enrolled sample, VR was not expected to lead to improvements in vestibular reflexes. In fact, it seems rather to act as a facilitator for improving a compensation strategy based on the enhancement of the vestibular network and sensory–motor integration for managing a correct trade-off between stability and advancement during gait. 

Finally, a limitation concerning VOR training is acknowledged, as we used only active head movements, while new rehabilitative strategies should also include passive, unpredictable head movements.

## 6. Conclusions

Our results provide novel evidence that combining VR with conventional neurorehabilitation could be a suitable complementary strategy for improving smoothness of gait and activities of daily living in patients with sTBI and that instrumental assessment could be used as an additional measure to quantify the efficacy of neurorehabilitation treatments.

## Figures and Tables

**Table 1:** Demographic and clinical characteristics at baseline (mean ± SD); VRg = vestibular rehabilitation group; CRg = conventional rehabilitation group; ABC Scale =Activities-Specific Balance Confidence Scale ranging from 0 (no confidence) to 100 (complete confidence); BBS = Berg Balance Scale, ranging from 0 (lowest level of function) to 58 (highest level of function); CB&M = Community Balance and Mobility Scale ranging from 0 (complete inability to perform the tasks) to 96 (the most successful completion of the tasks possible); CIQ = Community Integration Questionnaire ranging from 0 to 29 (high scores represent greater independence and community integration); DGI = Dynamic Gait Index ranging from 0 (lowest level of function) to 24 (highest level of function); DHI = Dizziness Handicap Inventory, ranging from 0 to 100 (the higher the score, the greater the perceived handicap due to dizziness). Student’s *t*-tests and a chi-square test were used to test group similarity at baseline.

		VRg (#15)	CRg (#15)	*p* Value
Age (years)		34.7 ± 12.8	36.8 ± 12.9	0.663
Sex N (%)	Male	7 (46.7)	12 (80)	0.128
	Female	8 (53.3)	3 (20)	
Time since trauma (months)		11.6 ± 7.3	9.3 ± 6.1	0.364
ABC Scale		74.1 ± 11.8	72.6 ± 15.1	0.983
CIQ		12.3 ± 9.9	10.7 ± 3.3	0.708
DGI		16.9 ± 5.1	17.9 ± 4.5	0.678
BBS		47.7 ± 5.8	44.2 ± 10.6	0.519
DHI		34.5 ± 17.1	32.5 ± 15.5	0.546
CB&M		37.7 ± 24.2	33.7 ± 23.0	0.740

**Table 2:** Clinical Scale Scores. Mean ± SD; VRg = vestibular rehabilitation group; CRg = conventional rehabilitation group; ABC Scale = Activities-Specific Balance Confidence Scale; BBS=Berg Balance Scale; CB&M = Community Balance and Mobility Scale; CIQ = Community Integration Questionnaire; DGI = Dynamic Gait Index; DHI = Dizziness Handicap Inventory; the symbols * and ** indicate statistically significant differences with respect to T0 (*p* < 0.05 and *p* < 0.001, respectively).

	VRg				CRg			
	T0	T1	T2	T3	T0	T1	T2	T3
DGI	16.9 ± 5.1	20.1 ± 3.8 **	20.8 ± 3.4 **	21.6 ± 3.4 **	17.9 ± 4.5	20.3 ± 4.1 **	20.9 ± 4.0 **	21.6 ± 3.3 **
BBS	47.7 ± 5.8	49.8 ± 6.0 **	50.3 ± 5.3 **	51.1 ± 5.6 **	44.2 ± 10.6	47.9 ± 9.6 **	49.5 ± 8.3 **	49.2 ± 8.5 **
DHI	34.5 ± 17.1	20.9 ± 12.9 **	20.9 ± 18.1 *	17.8 ± 17.4 *	32.5 ± 15.5	22.2 ± 12.7 *	16.2 ± 13.3 *	24.0 ± 22.0 *
CB&M	37.7 ± 24.2	47.2 ± 27.6 **	48.1 ± 24.8 **	48.8 ± 25.3 **	33.7 ± 23.0	39.0 ± 20.2	47.4 ± 25.9	49.7 ± 24.7 *
ABC	74.1 ± 11.8	-	-	84.2 ± 15.1 *	72.6 ± 15.1	-	-	76.1 ± 25.3
CIQ	12.3 ± 9.9	-	-	12.9 ± 4.4 *	10.7 ± 3.3	-	-	10.5 ± 4.2

**Table 3:** F8WT parameters divided into three subdomains of interest (i.e., spatio-temporal, stability, smoothness). Mean ± standard deviation values are reported for both VR and CR in the four assessment sessions. The presence of main effects of the session is highlighted, indicating the two sessions where statistical significance was found (e.g., “03” stands for a difference between T0 and T3).

F8WT Parameters		VR				CR				Main Effects
		T0	T1	T2	T3	T0	T1	T2	T3	
**ST**	SF (steps/s)	0.68 ± 0.16	0.78 ± 0.15	0.76 ± 0.12	0.81 ± 0.11	0.67 ± 0.18	0.70 ± 0.11	0.74 ± 0.13	0.75 ± 0.16	**03**
	WS (m/s)	0.70 ± 0.37	0.83 ± 0.36	0.84 ± 0.35	1.00 ± 0.26	0.62 ± 0.30	0.70 ± 0.22	0.83 ± 0.26	0.82 ± 0.35	**02, 03, 13**
**Stability**	RMS-P_ML_ (adim)	1.05 ± 0.31	1.01 ± 0.23	0.93 ± 0.18	0.86 ± 0.22	1.08 ± 0.34	0.92 ± 0.21	0.95 ± 0.21	0.95 ± 0.18	**03**
	RMS-T_AP_ (adim)	0.77 ± 0.36	0.67 ± 0.26	0.64 ± 0.26	0.55 ± 0.21	0.79 ± 0.34	0.71 ± 0.35	0.61 ± 0.23	0.67 ± 0.19	**02, 03**
	RMS-T_ML_ (adim)	1.04 ± 0.48	1.03 ± 0.50	1.06 ± 0.48	0.86 ± 0.26	0.97 ± 0.34	0.80 ± 0.21	0.75 ± 0.16	0.78 ± 0.17	**02, 03**
	RMS-T_mag_ (adim)	1.71 ± 0.48	1.63 ± 0.47	1.63 ± 0.45	1.46 ± 0.25	1.64 ± 0.37	1.50 ± 0.29	1.40 ± 0.19	1.45 ± 0.16	**02, 03**
	RMS-H_AP_ (adim)	0.83 ± 0.38	0.74 ± 0.34	0.61 ± 0.21	0.58 ± 0.21	0.86 ± 0.44	0.78 ± 0.38	0.63 ± 0.17	0.71 ± 0.27	**03**
**Smooth**	LDLJv_AP_ (%)	−4.58 ± 0.38	−4.25 ± 0.57	−4.25 ± 0.43	−4.25 ± 0.41	−4.60 ± 0.43	−4.43 ± 0.43	−4.25 ± 0.33	−4.37 ± 0.29	**02, 03, 12**
	LDLJv_ML_ (%)	−4.50 ± 0.34	−4.09 ± 0.44	−4.06 ± 0.23	−4.11 ± 0.53	−4.27 ± 0.42	−4.15 ± 0.28	−4.07 ± 0.34	−4.13 ± 0.32	**02**
	LDLJv_CC_ (%)	−3.72 ± 0.42	−3.35 ± 0.49	−3.24 ± 0.28	−3.39 ± 0.44	−3.62 ± 0.45	−3.63 ± 0.44	−3.46 ± 0.43	−3.42 ± 0.34	**01, 02, 03**

## Data Availability

The data presented in this study can be made available on request from the corresponding author.
